# Potential role of microRNAs in pancreatic cancer manifestation: a review

**DOI:** 10.1186/s43046-022-00127-2

**Published:** 2022-06-20

**Authors:** Lisa Kabiraj, Atreyee Kundu

**Affiliations:** Department of Microbiology, Techno India University, EM-4, Sector-V, Salt Lake City, Kolkata, 700091 India

**Keywords:** Pancreatic cancer, MicroRNA, miR biogenesis, Tumorigenesis, Biomarker, Genetic network, Prognosis, Therapeutic target

## Abstract

Cancer cells are different from normal cells in regard to phenotypic and functional expression. Cancer is the outcome of aberrant gene expression affecting various cellular signaling pathways. MicroRNAs (MiRs) are small, non-coding RNAs regulating the expression of various protein-coding genes post-transcriptionally and are known to play critical roles in the complicated cellular pathways leading to cell growth, proliferation, development, and apoptosis. MiRs are involved in various cancer-related pathways and function both as tumor suppressor and cancer-causing genes. There is a need for significant biomarkers, and better prognostication of response to a particular treatment and liquid biopsy could be useful to appraise such potential biomarkers. This review has focused on the involvement of anomalous expression of miRs in human pancreatic cancer and the investigation of miR-based biomarkers for disease diagnosis and better therapeutic selection.

## Background

This review focuses on the involvement of aberrant expression of microRNAs in human pancreatic cancer. These microRNAs serve as tumor biomarkers for disease prognosis and diagnosis. MicroRNAs are non-coding RNAs, actively participating both in cancer metastasis and as tumor suppressors. Since cancer shows heterozygosity, many different kinds of cells, proteins, and RNAs are present to indicate the abnormal formation. This review aims to highlight the role of certain important microRNAs associated with human pancreatic carcinoma, and their up- and downregulations have been found to be linked with tumor formation. Liquid biopsy, a non-invasive process followed by RNA profiling, can reveal the authentic microRNAs for each type of cancer. Pancreatic cancer is a deadly disease and requires early detection and treatment. This review targets for a better investigation of these non-coding RNAs present in the exosomes, resulting in epithelial mesenchymal transition (EMT). This branch of RNA biology needs to develop microRNA-based biomarkers and miR-based inhibition of tumor-causing genes using antisense oligonucleotides (ASOs) and certain specific drugs. The current issue is to bring out the microRNAs causing gene silencing for both cancer development and tumor suppressors. The early detection of such carcinomas can be fulfilled by miR-based biomarkers. There are certain miRs working as tumor suppressors and hence could be selected to lower the growth of tumor. It is effective against chemoresistance, by administering miR ASOs such as miR-221 ASOs and miR-21 in pancreatic ductal adenocarcinoma (PDAC). Continuous research on this area needs to be done for bringing up better therapeutic selection for better survival and disease-free population.

## Introduction

The identity of normal cells is maintained by regulative proliferation and differentiation. The approach of biology is to know how these cells gain and keep up their normal function. Cancer is the outcome of uncontrolled, proliferative, and incorrect growth of normal cells and ultimately transforming into abnormal. In cancer, the typical tissue morphology and plasticity are lost. The essential traits of malignancy include supported angiogenesis, by passing apoptosis, infinite replicative potential, inconsiderate to antigrowth signals, self-efficiency in growth signals, tissue onslaught, and metastasis. One remarkable characteristic of cancerous cell is defective regulatory pathways that alleviate the directed proliferation and differentiation of normal cell homeostasis. It is quite transparent that the expression of genes is altered in cancer cells [1].

Pancreatic cancer has caused pronounced health threats and the fourth leading root of cancer-associated deaths all over the globe. Therefore, there is a desperate requirement to interpret the molecular genetic mechanism implicating this carcinoma, along with genetic networks impacted during the malignant conversion process, so as to achieved finer detection, prognostic and diagnostic markers, and therapeutic goals that could assist in enhancing clinical care and therapeutic result for the diseased people [2].

There is a sore need to elaborate the molecular machinery linked with the happening, growth, progression, therapeutic resistance, and metastasis of this deadly disease. Several reports have revealed that the invasive properties of many kinds of cancers and in pancreatic cancer are correlated with the EMT [3]. It is the primary process by which epithelial cells lose their phenotypes and gain mesenchymal phenotypes. This transition to a mesenchymal state overlooks certain important properties of epithelial cells such as cell-cell adhesion and cell polarity. Partial EMT is often observed in vivo. The mesenchymal phenotype is regarded “metastable,” while the epithelial phenotype is considered as stable and proficient of colonization. EMT is regulated by complex genetic networks entailing epigenetic modifications, transcription factors, and transcription regulators. EMT-inducing transcription factors including SNAIL (Zinc finger protein SNAIL), Twist (Twist basic helix-loop-helix transcription factor 1), and ZEB (Zinc finger-E-box binding homeobox 1) and transcription regulators like microRNA (miRNA) expressions regulate EMT. SNAIL and ZEB are robust epithelial EMT repressors, and the loss of these transcription factors in pancreatic cell cannot avert pancreatic cancer [4].

An early asymptomatic malignancy can be detected with the help of biomarkers and can also bring out the prediction of response to selective treatment that will be extremely helpful for the patients. The extensive use of carcinoembryonic antigen (CEA) and carbohydrate antigen 19-9 (CA19-9) also have limitations in clinical practice. A single biopsy sample is not enough to focus on tumor progression and metastasis. We need biomarkers that could help in tumor manifestation and to stipulate the response to treatment and recurrence of tumor. Additionally, uncomplicated and repeated sampling should be the most essential feature of these biomarkers. Liquid or fluid biopsy is a procedure to diagnose cancer by tracing the circulating tumor cells (CTCs), microvesicles for instance exosomes carrying proteins, and nucleic acids and cell-free circulating nucleic acids (CFNAs), which are generally released into the blood from the metastatic tumors. Unlike other invasive biopsy methods, liquid biopsy is a non-invasive procedure for tumor diagnosis. It gives real-time findings and provides a successful prognosis. In this review, we have discussed the roles of microRNAs in pancreatic cancer manifestation and the efficient biomarkers for this lethal disease prognosis [5–7].

## Non-coding, naturally synthesized RNAs

MiRs are conserved, small, endogenously synthesized, non-coding RNAs of about 17–25 long stretch of nucleotides. These miRs control the gene expression post-transcriptionally via either degradation of various messenger RNAs (mRNAs) or translational repression. It has been announced that expression patterns of miRs are more in illustrating as pathognomonic tumorigenic events as compared with mRNAs for human cancer [2, 8].

MicroRNAs were first discovered in roundworm, *Caenorhabditis elegans*, in 1993. It is observed that a gene named *lin-4* important for *C. elegans* development does not code for any protein, but this gene negatively regulates the translation of *lin-14*. This indicates the crucial regulatory functions of miRs in development, differentiation, programmed cell death (apoptosis), stress response, and proliferation. The key role of miRs is very clear now as universal regulators of gene expression and in targeting various cellular pathways [9, 10].

## Biogenesis of miRNA molecules and mode of action

**Fig. 1:**
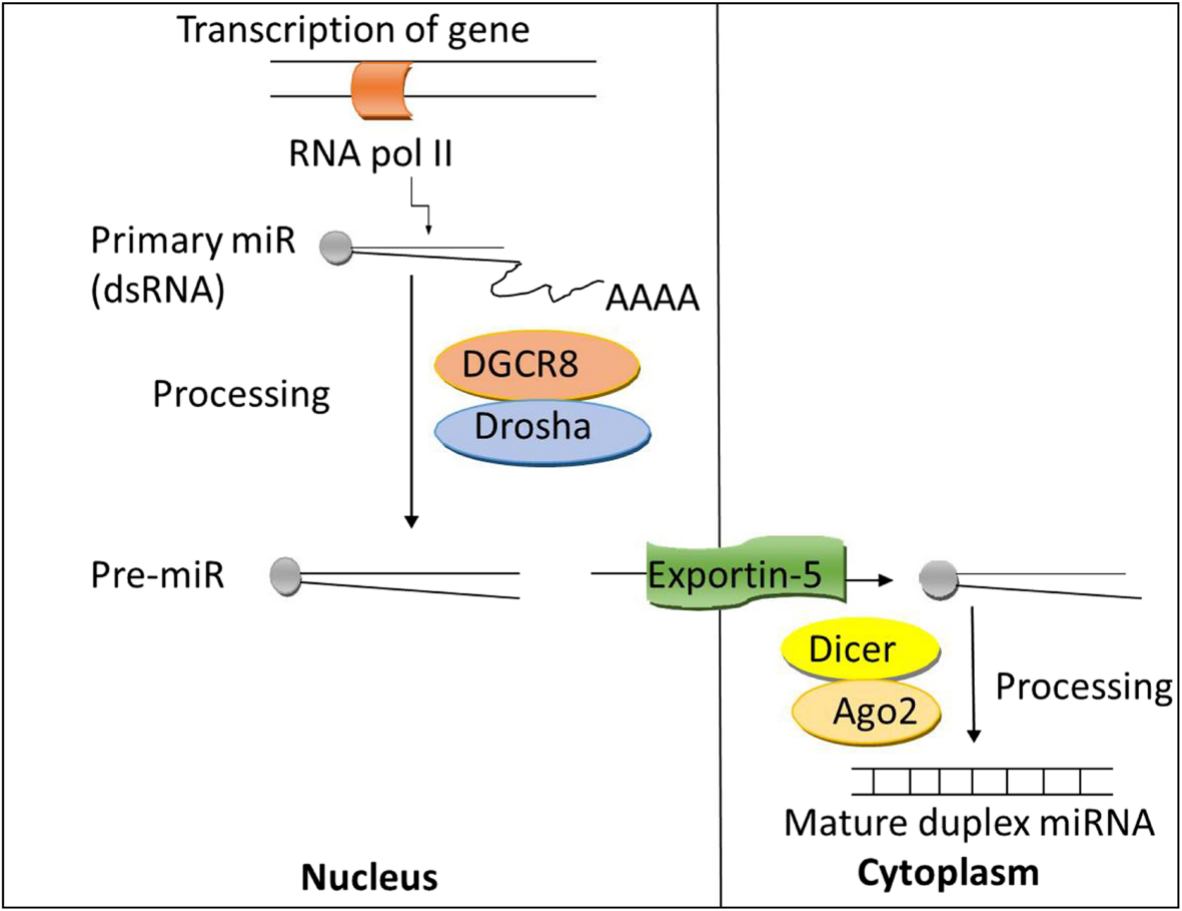
Overview of the miR biogenesis

**Fig. 2:**
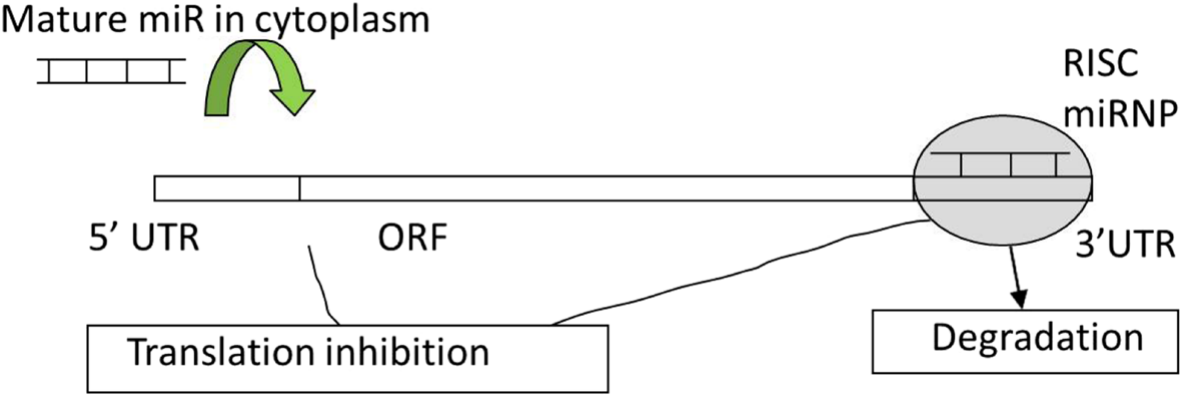
Mode of action of miR. RISC makes reference to the RNA-induced silencing complex

In the human genome, about 30% of protein-coding genes are controlled by miRs. Most of the miR genes are intragenic (found within the gene—70%), but some are restricted in gene desert areas (absence of protein coding genes—30%) as independent transcription units [11, 12]. Synthesis of miRs requires specific steps. The genes for miRs are localized in the nucleus of the cell. The long transcripts of primary miRs have a characteristic structure that helps to identify them and also prognosticate the target genes they might control. The effective form of a miR is generally 21 or 22 nucleotides in length. These short miRs are produced by two cleavage reactions that occur in long transcripts having secondary structures using two different RNase III endonucleases in the cell. These are naturally occurring molecules in the cell. At first, the gene in the nucleus is transcribed by RNA polymerase II into the long primary transcript, named primary miRNA (PRI-MIR) (Fig. 1). This process is followed by cleavage of pri-miR into 70–100 nucleotides having a hairpin loop structure by an RNase III endonuclease “Drosha” and DGCR8 found in the nucleus. This is now termed as precursor miR (Pre-miR). Then, the Pre-miRs are exported by the nuclear pore complex from the nucleus into the cytoplasm for further processing by “Dicer.” This export is mediated by GTP-dependent Exportin-5 protein. Pre-miRs are again modified by another enzyme, RNase III endonuclease Dicer and Ago2 in the cytoplasm into 17–22 nucleotides, resulting in the formation of double-stranded mature miRs. The single-stranded miRs are integrated in the multiprotein RNA-induced silencing complex (RISC) and instruct the complex to regulate the target mRNAs by complementary base pairing with the 3′ untranslated region (3′UTR). The 5′ untranslated region (5′UTR) is also called the “miRNA seed region” containing nucleotide 2–8 base pairing with them RNA complementary sequence in the 3′ untranslated region. If the complementarity is partial between the seed region and the 3′ untranslated region of target transcript, it inhibits translation, whereas perfect complementarities result in the degradation of mRNA (Fig. 2) [13–17].

## Micro RNAs as tumor suppressors

### The role of miR-6-1 and miR-15-a in chronic lymphocytic leukemia

Abnormal expression of miRs has been revealed in many human cancers. Studies have shown the crucial role of miRs in tumorigenesis. The target of miRs is those genes that are involved in cell cycle progression or programmed cell death. In many different types of cancers, the genes for miRs are deleted or amplified, relying on the chromosomal rearrangement. It was first observed that the most common appearance of adult leukemia in the hemisphere was the B cell chronic lymphocytic leukemia (CLL). This disease was due to the deletion of chromosome region 13q14. This genomic region contains miR15-a and miR-16-1 genes. These two miRs have the potential to trigger programmed cell death by downregulating the anti-apoptotic *bcl-2* gene. The expression of *bcl-2* is very high in CLL and results in the survival of malignant cancer cells. Binding sites for miR-15a and miR16-1 are observed in the 3′ untranslated region of *bcl-2*, and the co-expression of these miRs downregulates the 3′ untranslated region of *bcl-2*. It is noticed that the expression of miR-15-a and miR-16-1 is totally eroded or diminished in the case of CLL. Studies have also revealed that the defective miR biogenesis machinery leads to the aberrant expression of miRs in cancer cells [18–20].

### Different miRs acting as tumor suppressors

The downregulation of miR-145 and miR-143 is observed in colorectal tumors. These miRs are also downregulated in breast carcinomas and breast cancer lines [21, 22]. The gain of function mutation in miRs promotes tumorigenesis. Expressions of miRs are modified by epigenetic mechanisms, for example, histone methylation and histone acetylation. Abnormal epigenetic regulation has been detected in different human cancers [23].

## Exosomal miRs as liquid biopsy marker

The deregulation of exosomal miRs takes important parts in many different kinds of cancers. Exosomes are small membrane-bound vesicles of approximately 30–100 nm in diameter originated from endosomes [24]. These endosomes are released by cancer cells into the extracellular surrounding where they play a crucial role in intercellular communication, containing RNA, DNA, and proteins. Exosomes released from the cancer tissues are diluted with exosomes discharged by the normal tissues in the bloodstream. Therefore, substantial effort has been given for detecting proteins that are more particularly expressed on tumor-acquired exosomes. It has been informed that the combination of magnetic beads and anti-epithelial cell adhesion molecules (ANTI-EpCAM) could be used to isolate this subgroup of exosomes, and Ep-CAM-positive exosomes are more remarkably secreted by epithelial tumor cells including pancreatic ductal cancer [25]. Mir-155 and mir-1246 are important biomarkers of pancreatic cancer, and they have been seen to commit to gemcitabine resistance. Certain miRs such as miR-99 and miR-125b family members comprising miR-99b, miR-100, and miR-99a have been ascribed oncogenic functions, controlling the progression of pancreatic cancer, helping as prognostic markers and forecaster of chemo-responsiveness. Contrarily, some miRs including miR-34a, miR-148a, miR-200a, miR-200b, and miR-200c function as tumor suppressors in pancreatic ductal cancer with a prognostic impact that chiefly inhibits EMT. Authors could demonstrate that this serum miR-200 has prognostic and diagnostic capability. MiR-200b and miR-200c obtained from circulating exosomes have appeared to have both prognostic and diagnostic values in many other types of cancers such as ovarian cancer, lung cancer, prostate cancer, melanoma, and colon cancer [26–28].

## Deregulated expression of different miRs in pancreatic cancer

Many different expression profile studies have signified the deregulation of the expression of miRs in pancreatic tissues and cell lines. The study of the expression profiles has been presented to show obliging hallmarks for distinctive diagnosis of pancreatic cancer from chronic inflammatory pancreatic disease (Table 1) [29]. The most common kind of pancreatic cancer appears from pancreatic ductal cells and is termed pancreatic ductal adenocarcinoma (PDAC) [30]. The tissue samples from PDAC, chronic pancreatitis, and normal pancreas patients were taken for miR expression profiling. A vast examination of miR expression profiles using microarray technology disclosed that some miRs, such as miR-143, miR-29c, miR-148b, miR-150, and miR-96, were distinctively present in PDAC as well as chronic pancreatitis samples, while the expression of some miRs, such as miR-196b, miR-203, miR-196a, miR-210, miR-222, miR-210, miR-216, miR-375, and miR-217, were observed changed only in pancreatic ductal carcinoma samples [23]. The authors deduced that miR-196a and miR-217 expression profiles could differentiate pancreatic ductal cancer from chronic pancreatitis and normal pancreas. In a study, it is found that the downregulation of miR-375 and miR-148a,b together with the over-expression of miR-21, miR-155, miR-221, miR-155, and miR-181a,b,c,d can discriminate PDAC from pancreatitis [31]. The author concluded that in a third study with 17 pairs of pancreatic cancer/normal tissues and 10 cell lines of pancreatic cancer, the increased expression of miR-190, miR-196a, miR-222, miR-15b, miR200b, miR-95, and miR-221 were found [32]. Fascinatingly, the upregulation of both the miR-21 and miR-155 both was observed very high in the intraductal papillary neoplasm (IPMN) in contrast to matched controls. It is clear that the abnormal expression of these miRs is prior to the multi-stage progression of the disease [33–36].

**Table 1:** Deregulated miRs involved in PDAC

MiR	Expression profile	References
miR-21	Upregulated	[32]
miR-31	Upregulated	[37]
miR-96	Downregulated	[30]
miR-99	Upregulated	[32]
miR-107	Upregulated	[32]
miR-143	Upregulated	[37]
miR-146	Upregulated	[32]
miR-148a	Downregulated	[32]
miR-148b	Downregulated	[32]
miR-155	Upregulated	[33]
miR-181b	Upregulated	[32]
miR-190	Upregulated	[34]
miR-196a	Upregulated	[34]
miR-200c	Upregulated	[35]
miR-205	Upregulated	[32]
miR-217	Downregulated	[37]
miR-221	Upregulated	[35]
miR-222	Upregulated	[32, 34]
miR-375	Downregulated	[37]
miR-429	Upregulated	[35]

## Role of miRs in angiogenesis of PDAC

Cancer cells acquire new vessels by the process of angiogenesis. Many studies have revealed that numerous miRs are involved in PDAC angiogenesis. Particularly, the upregulation of miR-34 can affect biological processes, such as angiogenesis, cell cycle progression, apoptosis, and metastatic potential, but is downregulated in PDAC [23, 37].

The upregulated miR-410 can inhibit angiogenesis in PDAC. It has also been shown that the co-repression of both miR-21 and mir-210 has been associated to the repression of angiogenesis and targeting of invasion and migration of cancer cells. A further study has conveyed that hypoxia can potentially promote PDAC, angiogenesis, cell migration, and invasion. MiR-221 or mir-222 play a crucial role in the tumorigenicity of PDAC [38–41].

## Signaling pathways involved in PDAC

Multiple abnormally expressing miRs accumulate and trigger aberrant phenotypic alterations in malignant cells by targeting different signaling pathways. MiR-21 expression is significantly high in PDAC, and it negatively regulates the phosphatase expression and tension homolog 2 (PTEN), tissue inhibitor of metalloproteinase 3 (TIMP3), and programmed cell death 4 (PDCD4) proteins resulting in malignant transformation, facilitated invasion, and apoptosis inhibition [42]. The authors have investigated the 11 frequently upregulated miRs in PDAC, targeted 71 genes by those upregulated miRs, and detected them to be downregulated in PDAC microarray profiling studies. This is attained with the help of Ingenuity Pathway Analysis. Nf-kB represents a critical role in cancer cell proliferation [11, 43]. The authors have shown five miR targets, mainly RPS6KB1, FOXO1, CDKNIB, INSR, and PTEN, and each of these genes have active involvement in regulating cell proliferation and have been blamed to have important roles in many other cancers in humans (Table 2).

**Table 2:** Genes involved in the signaling pathways targeted by 11 over-expressed miRs in PDAC attained with Ingenuity Pathway Analysis (IPA) [12]

Signaling pathway	*p*-value	Ratio	Molecules
PTEN signaling	8.48E−05	5/123 (0.041)	RPS6KB1, INSR, CDKNIB, PTEN
PIEK/AKT signaling	1.66E−03	4/142 (0.028)	RPS6KB1, FOXO1, CDKNIB, PTEN
Insulin receptor signaling	2.20E−04	5/141 (0.035)	RPS6KB1, FOXO1, PPP1R11, INSR, PTEN

RPS6KB1 being a ribosomal protein S6 kinase activates growth factors, for instance, epidermal growth factor (EGF), platelet-derived growth factor (PDGF), and mechanistic target of rapamycin (mTOR) leading to cell growth. CDKNIB/p27 belongs to the Cip/Kip family of Cyclin-dependent kinase inhibitors (CDKI) and acts as a tumor suppressor protein because of having the capability to trigger cell cycle arrest and is detected inactivated in several tumor kinds. The PI3K/AKT pathway is directly linked to cell proliferation and cancer and inhibition of apoptosis. Transcription factors like FOXO control multiple processes such as cell cycle, apoptosis, and DNA repair and negatively regulate PI3K/AKT and Erk signaling pathways in malignant cells. PTEN tumor suppressor is inactivated in pancreatic cancer, and deletion of PTEN would lead to the deregulation of the Ras/Raf and PTEN/PI3K/AKT pathways, resulting in the initiation and progression of pancreatic cancer [11, 44]. The tumor suppressor, PTEN, suppresses the PI3K-AKT-mTOR pathway and can credibly be targeted by miR-221, miR-21, and miR-181a. MiR-21 is involved in cancer cell proliferation by inhibiting apoptosis and cell cycle arrest, and therefore, the inhibition of miR-221 can lead to invasion, migration, and uncontrolled proliferation of PDAC cells. Mir-181a is also involved in the migration of PDAC cells [45, 46].

The presence of over-expressed miRs can provide confidence to develop biomarker assays for the detection of disease and prognosis. Some downregulated miRs are tumor suppressors, such as downregulated miR-34a in pancreatic cancer is straightly trans-activated by p53 protein. The expression of miR-34a prompts apoptosis, negatively acts on cell cycle progression, angiogenesis, and DNA repair. The CpG methylation in the promoter of miR-34a results in transcriptional silencing has been demonstrated in malignant cells. Loss of miR-let-7 induces cancer cell proliferation by activating MAPK and K-Ras expression. It is rational to propose that different miRs can be associated in different stages of PDAC [47, 48].

## Regulation of EMT

**Fig. 3:**
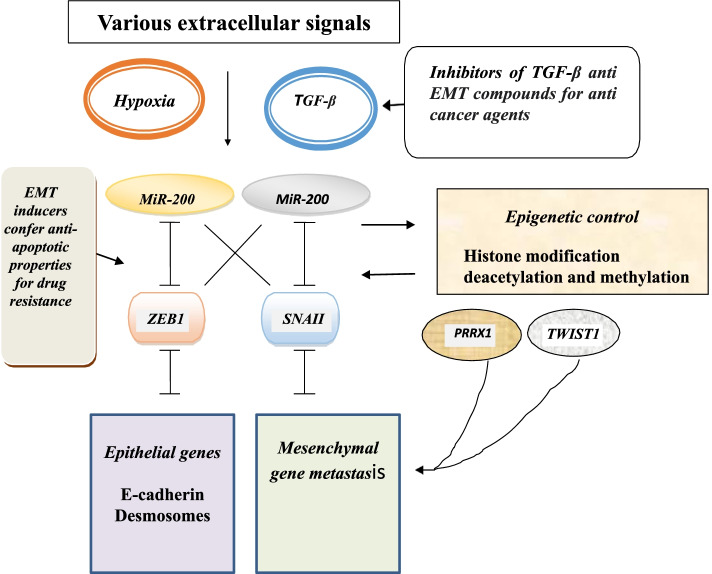
EMT controlling network: important sites discussing both chemoresistance and anti-EMT agents/representatives for therapy of cancer

EMT is modulated by a complicated network entailing transcriptional control, stability of protein, epigenetic modifications, subcellular localization, and alternative splicing [49, 50]. Certain pathways are important for a particular EMT event during the development of tumors, for example, differentiation, metastasis, and tumor formation. EMT transcription factors are considered rulers of these intricate networks (Fig. 3). Members of the superfamily TGF-β is regarded as a primary EMT inducer [4]. This signaling also triggers many other EMT-inducing pathways such as delta-Notch, wingless (WNT), and integrin signaling pathways. SNAIL and ZEB are considered epithelial suppressors rather than mesenchymal supporters [51]. Twist and PRRX are defined as powerful mesenchymal inducers. ZEB and SNAIL suppress the epithelial marker expression, for instance, claudins, occludins, and E-cadherin, which are downstream of this modulating loop. The family of miR-200 is inappropriately linked with both increased metastasis and decreased invasion, and the outcome is combined repression of Twist, SNAIL ½, and ZEB ½ but had no influence on metastasis. Consecutively, ZEB1 represses the expression of the miR-200 family, including miR-203 which inhibits stemness. This negative feedback calls attention to the role of EMT transcription factors in stemness and the correlation of maintenance of stemness and EMT regulation to pancreatic cancer. Additionally, in the SNAIL1-independent pathway, paired related homeobox 1 (PRRX1) is an EMT inducer and operates invasiveness together with TWIST1 [52]. The expression of the miR-200 family is also regulated by DNA methylation and histone demethylase [4].

## The role of miR-128 in pancreatic carcinoma

The expression of mir-128 may be suppressed at the transcriptional level and is associated with various kinds of cancers. It has been discussed that mir-128 is found to be decreased in pancreatic cancer while the adjacent non-cancerous tissue has a normal level of mir-128. The double minute (MDM) family proteins regulate tumor-suppressor protein p53 [53]. The authors have recognized that MDM4 is a p53-binding protein, and MDM4 knockdown can trigger embryonic lethality in mice [54]. The authors have conducted several assays, such as colony formation assay, colony formation assay, flow cytometry analysis of apoptotic cells, western blotting, and quantitative reverse transcription polymerase chain reaction, and finally concluded that mir-128 has a potential role in inhibiting pancreatic cancer proliferation by targeting MDM4 to trigger cell apoptosis [55].

## Discovery of miR-126 in pancreatic carcinoma

EMT is a crucial pace for pancreatic carcinoma as an arrival of metastatic malady. A large variety of signal transduction pathways and cytokines are associated in this multiplex process, but the whole sketch is still mysterious. EMT-triggering pathways could be targeted for a novel therapeutic plan in pancreatic carcinoma. The authors demonstrated for the first time the significant role of miR-126/ADAM9 in the suppression of metastatic growth of the disease. The straight interconnection between mRNA\ADAM9 and miR-126 was established by the 3′UTR assay by the authors.

## MiRs as biomarkers in PDAC

Biomarkers are the molecules mainly found in body fluids or tissues, and their presence indicates the condition of cells. The presence of significant miRs defines the onset or presence of disease in the body. These can be prognostic or diagnostic markers. The functional role of miRs in regulating multiple mRNA transcripts presents a great opportunity in developing both prognostic and diagnostic markers. Some findings have disclosed that miR-217 and miR-216 are present in PDAC cells. MiR-133a is absent in PDAC, and about 26 miRs are abnormally expressed in PDAC. Normal pancreas and chronic pancreatitis can be distinguished from PDAC by the over-regulation of miR-196a and downregulation of miR-217 [2, 29].

MiR-155 and miR-21 are over-expressed in PDAC, and levels of these two miRs can discriminate pancreatic cancer from benign lesions with an eminent accuracy as compared with standard pathology. miR-21 acts as a prognostic marker and can detect the risk of relapse in PDAC. It is important to detect at the initial stage of disease in order to provide the right treatment and care to the patients. MiR-based biomarkers can help in the early diagnosis of disease [56].

Some of the miRs have been observed to act as cancer-causing genes in pancreatic cancer, and these miRs are involved in tumor formation and metastasis. These oncogenes can intensify abnormal cell proliferation through repressing the genes which regulate important cell cycle transition. These miRs basically suppress the translation through destabilizing the mRNA. One of the upregulated miRs, miR-212, is responsible for cancer proliferation in PDAC cells. The cancer-causing role of miR-212 has been established through the transfection studies in PDAC cells employing miR-212 mimics/inhibitors, and miR-212 has been observed to target PTCH1. The expression of PTCH1 can be regulated by miR-212 resulting in promoting metastatic capability and PDAC transformation and progression. Another upregulated miR-221-3p is associated in PDAC. MiR-221-3p has amplified the multiplication and repressed the apoptosis of SW1990 cells [57].

## Therapeutic targeting of non-coding miRs in PDAC

Recent studies have shown that the miR delivery systems can be used to suppress PDAC. Polymeric nanoformulations delivering miR-150 and liposome-based nanoparticles carrying miR-143/miR-145 and miR-34a are capable of inhibiting tumor growth in PDAC both in vitro and in vivo [57, 58]. Mature miRs are targeted using ASOs to silence the upregulated genes responsible for tumor growth and also to target the over-expressed onco-miRs, which have proven the active roles of these onco-miRs in tumor proliferations. It is found both in vitro and in vivo that there is depletion in malignant cell proliferation, migration, and chemoresistance by administering miR-221 ASOs and miR-21 ASOs in murine pancreatic cell models. MiR-based therapeutics has been revealed to be safe and effective against diseases and infections. Some therapies based on miRs are now ongoing in cancer patients and hope that the advancement in technology will qualify the delivery of miRs through different delivery systems such as via liposomal nanoparticles.

The miR and ASOs combined with gemcitabine, a therapeutic drug for cancer, have a cooperative effect in delaying pancreatic cell growth [59,60].

## Concluding remarks

Cancer is a deadly disease, and pancreatic cancer has a very low survival rate. The loss of function mutation or downregulation of tumor suppressors promotes tumor growth. The downregulation of gatekeeper genes triggers cell proliferation and inhibits apoptosis and ultimately affects the normal tissue homeostasis. MiRs are involved in tumor development, proliferation, and metastasis. A detailed study on the presence of specific miRs and their functions in PDAC is necessary in order to provide a better understanding and select biomarkers for disease identification. The use of miRs together with conventional therapeutic agents can be a hopeful proposal for solving drug resistance in PDAC. MiR-based therapeutics is safe for humans, and the development of technology is likely to upgrade these therapeutic administrations. MiR can provide both prognostic and diagnostic information and selection for better treatment.

## Future prospect

MicroRNAs are the master of gene modulators, and the research data shows the potential role of miRs in manifesting cancer even before the severity of the disease. This branch of RNA biology needs more research for establishing proper biomarkers for cancer and also miR-based inhibition of certain tumor-inducing genes and proteins. Pancreatic cancer needs proper prognostic, diagnostic, and therapeutic strategies in overcoming the stress and lethality of the disease. Continuous research could help in detecting the cause and expression of genes and their inhibition by targeted miRs. Our expectation is to provide an early diagnosis that could be fulfilled with the help of liquid biopsy, and we believe that the existing data are enough to provide hope in disease management.

## Data Availability

All data generated or analyzed during this study are included in this published article.

## Abbreviations

MiRs: MicroRNAs
3′UTR: 3′ untranslated region
PDAC: Pancreatic ductal adenocarcinoma
mRNAs: Messenger RNAs
mTOR: Mammalian target of rapamycin
RISC: RNA-induced silencing complex
IPMN: Intraductal papillary neoplasms
ASOs: Antisense oligonucleotides
MDM: Double minute
EMT: Epithelial mesenchymal transition
CLL: Chronic lymphocytic leukemia
PTEN: Phosphatase and tensin homolog 2
